# Targeting SAA expression via siRNA mitigates preterm birth induced by maternal inflammation

**DOI:** 10.3389/fphar.2026.1749966

**Published:** 2026-01-16

**Authors:** Jun Lei, Yang Liu, Jin Liu, Anguo Liu, Kimberly Jones-Beatty, Elizabeth Ann L. Enninga, Courtney Townsel, Irina Burd

**Affiliations:** 1 Integrated Research Center for Fetal Medicine, Department of Obstetrics, Gynecology and Reproductive Sciences, University of Maryland School of Medicine, Baltimore, MD, United States; 2 Department of Pathology, Johns Hopkins University School of Medicine, Baltimore, MD, United States; 3 Maternal Fetal Medicine Division, Department of Obstetrics and Gynecology, Mayo Clinic, Rochester, MN, United States

**Keywords:** maternal inflammation, P2X7R, preterm birth, serum amyloid A, siRNA

## Abstract

**Introduction:**

Placental inflammation is a major contributor to preterm birth (PTB), and there are currently few targeted strategies to prevent PTB and its associated adverse neonatal outcomes. Serum amyloid A (SAA), particularly the isoforms SAA1 and SAA2, are well-recognized inflammatory markers, but their functional roles in placental inflammation remain poorly defined.

**Methods:**

Using a translational mouse model of sub-chronic maternal inflammation, we investigated the immune mechanisms and therapeutic potential of siRNA-mediated targeting of Saa2 (siSaa2). Placental expression patterns of SAA2 were examined *in vivo*, and macrophage responses to extracellular SAA2 were modeled in vitro using RAW264.7 cells to assess downstream P2X7R-dependent signaling and functional outcomes.

**Results:**

SAA2 is primarily induced in placental trophoblast and endothelial compartments during inflammation, where it acts as an extracellular inflammatory mediator. *In vitro*, we model macrophage responses to extracellular SAA2 using RAW264.7 cells to examine downstream P2X7R-dependent signaling and functional outcomes. Maternal administration of siSaa2 improved PTB rates, placental morphology and fetal brain development. Additionally, SAA1 and SAA2 exhibited distinct expression patterns in the mouse and human placenta.

**Discussion:**

These findings identify placental SAA2 as a key inflammatory mediator in inflammation-associated PTB and demonstrate that targeted silencing of Saa2 represents a potential therapeutic strategy to mitigate placental injury and adverse fetal outcomes.

## Introduction

Preterm birth (PTB), defined as parturition prior to 37 weeks, is a leading cause of neonatal morbidity and mortality, and it is often associated with maternal infection and inflammation. Fifteen million babies are born prematurely worldwide, contributing to more than 1 million deaths annually among children under 5 years (Blencowe et al., 2013a; Blencowe et al., 2013b; Romero et al., 2014). One tenth of pregnancies in the United States and 5%–18% of births globally are associated with PTB (Bronstein et al., 2018). The short- and long-term adverse outcomes of PTB impose substantial health and economic burdens on families and society (Waitzman et al., 2021). However, there are limited therapeutics available to prevent PTB and the poor sequelae in the premature offspring due to its complex etiology and our incomplete understanding of its underlying mechanisms (Coler et al., 2021; Para et al., 2021; Francis et al., 2019; Tedesco et al., 2020; Goldenberg et al., 2008; Simmons et al., 2010).

Serum amyloid A (SAA) is a representative acute phase protein (APP) (Zhang et al., 2019; den Hartigh et al., 2023) triggered by infection, tissue damage and inflammation (Uhlar and Whitehead, 1999; Yamada, 1999). Compared to the elevation of other APPs under such circumstances, SAA expression has been reported to occur more quickly and to be more sensitive to PTB than other forms of infectious diseases (P et al., 2003; Cekmez et al., 2013; Koseoglu et al., 2014; Ibrahim et al., 2021; Connolly et al., 2010). Studies have shown that isoforms of serum amyloid A (namely, SAA1-4) exhibit species-, strain-, tissue- and cell-specific expression patterns (Uhlar and Whitehead, 1999; Webb, 2021; Meek and Benditt, 1986; Upragarin et al., 2005; de Beer et al., 1996; Mori et al., 2014a; Yamamoto et al., 1986; Marhaug et al., 1997; Rygg, , 1996). These isoforms vary in their response to inflammatory stimuli in both humans and other animal species (Webb, 2021; Meek et al., 1994; Larson et al., 2003; Chait et al., 2021). Notably, SAA1 and 2 are highly homologous with regard to their amino acid sequences. SAA does not have its own receptors to induce proinflammatory cascades (den Hartigh et al., 2023; Sack, 2018; Niemi et al., 2011), but rather has varied levels of affinity for binding partners, including five receptors and four proteins (Fan et al., 2019). As such, SAA acts as a cytokine-like factor in its regulation of cell‒cell communication (Sack, 2018), indicating a potential causal role in inducing inflammatory responses.

In our previous study, we employed a mouse model of acute intrauterine maternal inflammation using high doses of lipopolysaccharide (LPS, a membrane component of Gram-negative bacteria) near term, which was expected to induce strong immune responses. Through this model, we identified SAA2 (corresponding to human SAA1 in terms of nomenclature) as a placenta-targeted molecule associated with PTB (Liu et al., 2022a), similar to another published finding regarding the unique feature of mouse SAA2 in the plasma (Yang et al., 2009). We further demonstrated that selective silencing of *Saa2* expression, using a small interfering RNA (siRNA)-based therapeutic approach, reduced the occurrence of PTB in states of acute maternal inflammation, supporting the notion that placental SAA2 may be a potential therapeutic target for PTB.

It is important to note that inflammation models varying in mediators, approaches and dosages lead to distinct differences in immune responses (Lee et al., 2019; Dong et al., 2019; Lei et al., 2017; Sones and Davisson, 2016). The LPS dose used in the acute intrauterine model is approximately 4000 times higher than the dose sufficient to elicit symptoms in humans (Schwarz et al., 2014; Szermer-Olearnik and Boratynski, 2015). Additionally, the markedly different systemic SAA levels observed in acute vs. chronic inflammatory conditions suggest distinct underlying mechanisms (Ehlting et al., 2021). Therefore, to investigate the role of SAA and better understand the mechanisms of preventing PTB while recapitulating human diseases, a more clinically relevant animal model of maternal inflammation is needed.

In this study, we utilized our established mouse model of sub-chronic maternal inflammation, administering interleukin-1 β (IL-1β), a key endogenous mediator of inflammation (Morgan, 2016; Faye-Petersen, 2008), to explore changes of SAA isoforms in the placenta. We evaluated the impact of siRNA treatment on immune responses and signaling pathway using P2X7R-engineered mutant mouse strain. Finally, we examined the expression patterns of SAA isoform expression in the human placenta.

## Materials and methods

### Ethics statement

This study was carried out in accordance with recommendations in the Guide for the Care and Use of Laboratory Animals of the National Institutes of Health. The protocols were approved by the Institutional Animal Care and Use Committee and Department of Health Safety Environment at Johns Hopkins University and University of Maryland.

### Animal preparation

CD-1 dams were obtained from Charles River Laboratories (Wilmington, MA). C57BL/6J mice and P2X7R knockout (KO) mouse strains were sourced from The Jackson Laboratory (Bar Harbor, ME).

Time-pregnant dams were subjected to an established model of sub-chronic maternal inflammation, as previously described (Chudnovets et al., 2020). From embryonic (E) days 14–17, 0.5 μg of mouse recombinant IL-1β (Sigma‒Aldrich, St. Louis, MO) in 100 μL PBS or 100 µL of PBS alone was intraperitoneally injected (IP) daily. A set of dams were given si*Saa2* (#4390771, ThermoFisher Scientific, Halethorpe, MD) at a dose of 74 nmol/kg dailyintravenously (IV) through the tail vein (Liu et al., 2022b; Liu et al., 2021; Na et al., 2021; Yang Liu et al., 2022; Heidel et al., 2007; Wrobel et al., 2021) (equivalent to 1 mg/kg). The siRNA sequences (5′→3′) used were as follows: sense strand, CUG​GAA​AGA​UGG​AGA​CAA​ATT; antisense strand, UUU​GUC​UCC​AUC​UUU​CCA​GCC. Dams were attributed to four groups: PBS+PBS (CD-1, *n =* 30; C57BL, *n =* 5, P2X7R KO, *n =* 5), IL-1β + PBS (CD-1, *n =* 47; C57BL, *n =* 7, P2X7R KO, *n =* 6), IL-1β + si*Saa2* (CD-1, *n =* 30) and PBS + si*Saa2* (CD-1, *n =* 10).

A set of 16 CD-1 dams were utilized, evenly allocated across the groups in the acute maternal inflammation study. Dams at E17 were placed under isoflurane anesthesia (Baxter Healthcare, Deerfield, IL) following a mini laparotomy. A dose of 25 μg of LPS (from *E. coli* O55:B5, Sigma Aldrich) in 100 μL PBS or PBS alone was injected between the first and second embryos of the lower right uterine horn. Routine laparotomy closure was performed, and dams were returned to their cages individually followed si*Saa2* administration 1 hour later.

Birth before E19 or absence of a fetus at an implantation site was categorized as PTB. Fetal brains and placentas from the first four gestational sacs of the right uterine horn were harvested at E18. Small or discolored fetuses in the uterus were categorized as abortion.

### Human study participants

Patients who delivered at term (>37 weeks) at the University of Maryland Medical Center whose respective placental specimens have been sent for pathological analysis were included in the study (*n* = 5). Our inclusion criteria were 18–45 years old with singleton. Patients with fetal structural anomalies and aneuploidies, infectious diseases, and any other diseases were excluded. This study was approved by the Institutional Review Board of the University of Maryland, Baltimore, under the approved protocols of HP-00100457 and HP-00106274.

### Cell culture and treatment

RAW264.7 macrophage mouse cell lines were obtained from the American Type Culture Collection (Manassas, VA, ATCC TIB-71). Cells were grown in Dulbecco’s Modified Eagle’s Medium (DMEM, ATCC) supplemented with 10% of FBS (Gibco, Waltham, MA) and 1% of pen/strep (Gibco) and incubated at 37 °C 5% CO_2_ humidified incubator. The culture medium was changed every 2 days. Cells were cultured with the addition of mouse SAA2 protein (CUSABIO Technology, Houston, TX) at 100–1,000 ng/mL or LPS (1 μg/mL) based on published *in vitro* studies (Niemi et al., 2011; Bjorkman et al., 2010; Gaiser et al., 2021) and pilot experiments showing macrophage activation within this range. Cells and culture supernatants were stored at −80 °C for further use.

### Enzyme-linked immunosorbent assay (ELISA)

Cytokine levels, IL-1β, IL-6 and TNFα, in the placenta and cell culture supernatant were performed using mouse ELISA Kits (Abcam, Branford, CT) according to the protocol provided by the manufacturer. Experiments were performed in triplicate. Assay sensitivity, defined as the lowest standard curve concentration, was confirmed to be met for all the values in each sample examined. Consequently, all values for each sample examined were above the limit of detection and within the reportable range of each assay. Concentrations quantified in supernatants were expressed in picograms per milliliter. For placental tissue, concentrations were expressed as fold change.

### Histochemistry and immunohistochemistry

Placentas and fetal brains were fixed in 4% paraformaldehyde (PFA) overnight at 4 °C followed by saturation in 30% sucrose for cryoprotection and storage. The specimens were cut at 20 µm thickness and mounted on positively charged slides using a Leica CM1950 cryostat (Leica Biosystems Inc., Deer Park, IL). Routine H&E staining was performed to evaluate the structural changes of placentas. Nissl staining was performed on fetal brains to evaluate cortical thickness and ventricle area. Images were taken under ×5 magnification using a Canon EOS Rebel (Tokyo, Japan).

For each animal, five areas of fields were selected. The straight-line tool of ImageJ software (v1.48, http://imagej.nih.gov/ij/, National Institute of Health) was utilized to measure the thickness of the fetal side of the central cut surface of the placenta and context of fetal brain. The fetal side of the placenta was identified by characteristic features of maternal/fetal blood vessels and anatomical structures, including the junctional zone and labyrinth. Cortical thickness was measured at the striatum level of each fetal brain, as previously described (Vermillion et al., 2017). An average of 10 measurements per specimen is presented. Quantification represents the average measurement from a single fetal brain for each dam.

For immunofluorescence staining, antigens were retrieved in boiling retrieval buffer for 20 min, followed by blocking in 10% goat serum and permeabilization with 0.5% Triton-X-100 in PBS. Placental sections were incubated with primary antibodies overnight at 4 °C. The primary and secondary antibodies that were used are described in Supplementary Table S1. The next day, sections were rinsed with PBS, followed by incubation with secondary antibodies for 3 h at room temperature. The sections were further stained with DAPI (4′,6-diamidino-2-phenylindole, 10236276001, Roche, Indianapolis, IN) for 2 min at room temperature followed by mounting with Fluromount-G (eBioscience, San Diego, CA, USA). Images were obtained using an Axioplan 2 Imaging system (Carl Zeiss, Thornwood, NY) from the same staining batch.

### Liquid chromatography-tandem mass spectrometry (LC-MS)

A standardized mass spectrometry-based quantitative proteomic profiling was performed using the TMT-18plex labeling technique. The workflow includes the preparation of tissue protein lysate, trypsin digestion, TMT-18plex labeling of tryptic peptides, fractionation of labeled peptides by reverse-phase UHPLC, LC-MS/MS analysis, database search, and data analysis.

### RNA extraction, immune array, and real-time quantitative polymerase chain reaction (RT‒qPCR)

Placentas were freshly frozen on dry ice, followed by long-term storage at −80 °C. RNA was extracted using a RNeasy Mini Kit (Qiagen, Germantown, MD). Two micrograms of RNA was applied for complementary (c) DNA synthesis in a 40 µL reaction using a Bio-Rad iScript™ cDNA Synthesis Kit (Bio-Rad, Hercules, CA). A TaqMan® Mouse ImmuneArray v2.1 (Thermo Fisher Scientific) was used to evaluate 96 immune markers. One hundred μL of 2× iTaq Super Mix (Bio-Rad) was diluted with 60 μL of water and 40 μL of prepared cDNA. Analyses were performed with the Quant Studio 12K Flex Real-Time PCR System (Thermo Fisher Scientific) for 40 cycles. Additional individual RT-qPCR was conducted for array validation and *Saa2*. The primers for *Saa2* (Mm.PT. 58.43376774), *Il1β* (Mm.PT.58.41616450), *Il6* (Mm.PT.58.10005566), and *β-actin* (Mm.PT. 39a. 22214843) were obtained from Integrated DNA Technologies. The primer for *18S* (catalog no. 4310893E) was obtained from Applied Biosystems. RT‒qPCR was performed with Universal Master Mix II (Thermo Fisher Scientific) on a CFX384 Real-Time PCR Detection system (Bio-Rad). Transcript levels were determined by normalizing the target gene threshold cycle (CT) value to the CT value of the endogenous housekeeping genes *β-actin* and *18S* (ΔΔCT).

### Western blotting (WB) and immunoprecipitation (IP)

Tissues were homogenized on ice in RIPA lysis buffer with proteinase inhibitor and phosphatase inhibitor cocktail 2 (Sigma‒Aldrich). The homogenized specimens were then placed on ice for 15 min and centrifuged at 14,000 rpm for 20 min at 4 °C. The resulting supernatants were collected for further experiments. Total protein was separated by sodium dodecyl sulfate‒polyacrylamide gel electrophoresis (SDS‒PAGE, Bio-Rad) using 4%–15% gels (Bio-Rad) and then transferred onto nitrocellulose membranes (Bio-Rad) using a semidry transfer device (Trans-Blot® Turbo™, Bio-Rad). The membranes were blocked with 5% bovine serum albumin (BSA, Sigma‒Aldrich) in Tris-buffered saline plus 0.1% Tween-20 (TBST) for 30 min at room temperature, incubated with primary antibodies in 5% BSA at 4 °C overnight, and then washed using TBST. The primary and secondary antibodies that were used are described in Supplementary Table S1. β-actin or GAPDH were used as quantitation control. Image acquisition was performed using a Li-Cor Odyssey Near Infra-Red System. ImageJ software was used to analyze all bands.

RAW 264.7 cells were harvested 24 h followed the treatment. Protein lysates were prepared using ice-cold RIPa buffer (Cell Signaling, Danvers, MA) supplemented with protease and phosphatase inhibitors. Lysates were pre-cleared with protein A/G agarose beads (Thermo Scientific, Cat#20423), and the supernatants were incubated with anti-P2X7 receptor antibody overnight at 4 °C with gentle rotation. Immune complexes were captured using protein A/G beads, washed extensively, and eluted in Laemmli sample buffer. Eluates, along with input and IgG control samples, were resolved by SDS-PAGE and transferred to PVDF membranes. Western blot analysis was performed using a phospho-Src (Tyr416) antibody to detect Src activation associated with P2X7R.

### Mouse genotyping

Approximately 2 mm mouse tail samples were cut and digested overnight at 55 °C with 0.5 mL of Genomic DNA Extraction Buffer (10 mM Tris-Cl pH 8.0, 10 mM EDTA pH 8.0, 50 mM NaCl, 0.5% SDS, and 0.5 mg/mL proteinase K). The resulting material was precipitated with isopropanol, washed in 70% ethanol and resuspended in TE pH 8.0. Eighty nanograms of total genomic DNA were then submitted to conventional PCR. P2X7R genotyping was determined by PCR amplification using the following oligonucleotide primers TCA​CCA​CCT​CCA​AGC​TCT​TC (WT), GCC​AGA​GGC​CAC​TTG​TGT​AG (KO), and TAT​ACT​GCC​CCT​CGG​TCT​TG (common). DNA sequencing of the PCR was performed by an E-Gel Power Snap electrophoresis device (Thermo Fisher, G8100) with 2% agarose gel (Thermo Fisher, V010920) according to the manufacturer’s instructions. The genotyping was confirmed by performing genomic sequencing of the respective amplicons (WT and heterozygous for each polymorphism).

### Cellular phagocytosis and cytotoxicity

Flow cytometry was applied to measure phagocytosis using an assay kit (Cayman, MI, USA) following the manufacturer’s instruction. Cell cytotoxicity was determined by measuring lactate dehydrogenase (LDH) activity in culture media using a commercially available kit (Biolegend, San Diego, CA). Briefly, the media were centrifuged at 3,000 rpm for 5 min. Cell-free culture supernatants were then incubated with the assay buffer and substrate mix in a new plate at room temperature. The absorbance at 490 nm was measured using a 96-well microplate reader (CLARIOstar LABTECH, Cary, NC). The background (spontaneous LDH release) value was set by measuring the media at time zero start from plating. Experiments were performed in triplicate.

### Flow cytometry analysis

Cells were stained with various surface markers at a 1:100 concentration in fluorescence-activated cell sorting (FACS) buffer for 30 min at 4 °C protected from light. OneComp eBeads (eBioscience) were used for single-color compensation controls. The antibodies and corresponding isotypes are listed in Supplementary Table S1. Data were acquired from an Attune NxT Acoustic Focusing Cytometer (Thermo Fisher Scientific) and analyzed with FlowJo 10 (FlowJo LLC, Ashland, OR). Debris and doublets were excluded by sequential gating on side scatter height versus side scatter area to delaminate singlets.

### Statistics and reproducibility

Data analyses were performed using GraphPad Prism 10 (GraphPad Software, Boston, MA). Grubbs’ test for outliers was conducted for each experimental group, and noted outliers were removed prior to analysis. PTB rate data were analyzed using Chi-square tests. Mass spectrometry-based quantitative proteomic profiling was analyzed using Student’s t-test to compare the PBS and IL-1β groups. Western blotting and RT‒qPCR gene expression data were analyzed using one-way analysis of variance (ANOVA) with Bonferroni *post hoc* tests for multiple comparisons of normally distributed data and Kruskal‒Wallis with Dunn’s multiple comparisons tests for nonparametric data. Results with a *p* value <0.05 were considered significant.

## Results

### Characterization of placental proteomic profiling and isoforms of SAA

**FIGURE 1:**
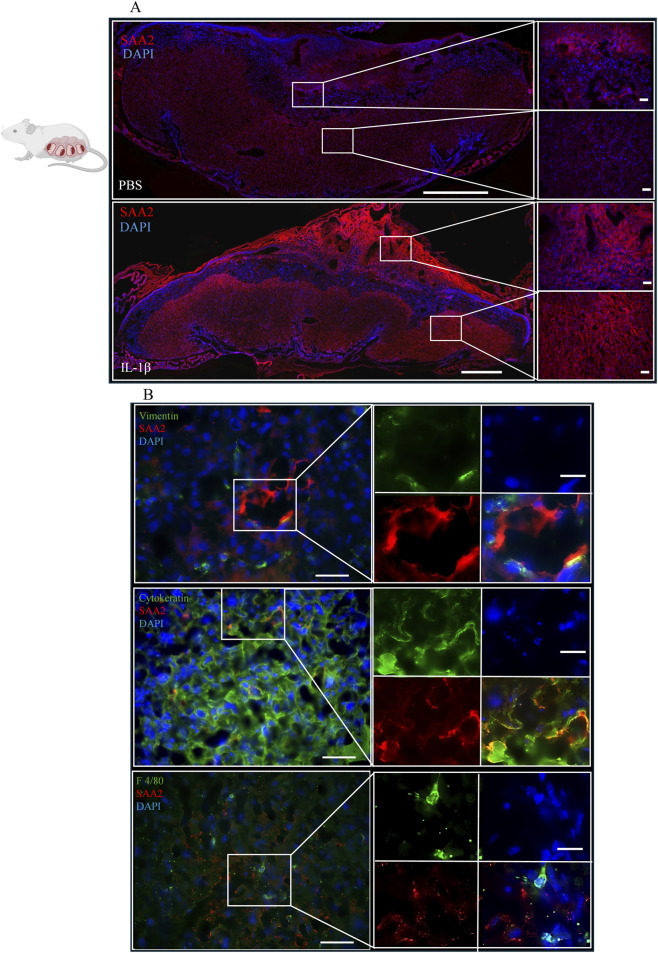
SAA2 expression pattern in the mouse placenta. From embryonic (E) day 14–17, CD-1 dams received IL-1β (0.5 µg) by intraperitoneal injection daily. At E18, placentas were harvested. **(A)** Representative images of SAA2 expression in the PBS and IL-1β groups (*n* = 3, 5 images per mouse examined). The right panels show high-magnification views of the areas outlined by white boxes in the corresponding left panels. Scale bar in the left panel, 1mm; in the right panel, 50 μm. **(B)** Fluorescent immune staining of SAA2 (red) and vimentin (green), an endothelial cell marker, or cytokeratin (green), a panel trophoblast marker, or F4/80 (green), a macrophage marker, in placentas. DAPI (blue) was used to label nuclei. Scale bar in left panel, 50 μm; in right panel, 10 μm.

The placental expressions pattern of SAA2 was examined using immunofluorescence staining. At E18, in the PBS group, SAA2 exhibited mild or absent expression in the decidua and labyrinth (Figure 1A, top panel). In response to IL-1β, SAA2 showed greater expression throughout the entire placenta (Figure 1A, bottom panel). We delineated the distribution and cell tropism of SAA2 in the placental labyrinth, where maternal-fetal exchange occurs. Cytoplasmic SAA2 expression was predominantly localized to vimentin+ endothelium (Figure 1B, top panel) and partially to cytokeratin+ trophoblasts (Figure 1B, middle panel) but was not observed in F4/80+ placental residential macrophages (HBCs, Figure 1B, bottom panel). This indicates that SAA2 is predominantly produced locally by parenchymal cells and vascular structures, with little to no expression detected in placental immune cells.

Since the SAA1 and SAA2 antibodies used were derived from the same host, SAA1 staining was performed on adjacent sections. SAA1 was primarily located in the mesometrial triangle, mainly around the vessel and the decidua in the PBS and IL-1β groups (Supplementary Figure S1A). In contrast, little or no SAA1 was detected in the labyrinth area (Supplementary Figure S1A), indicating a spatial distribution distinct from that of SAA2.

Our previous studies demonstrated an increase in SAA2 expression in the placenta in response to acute maternal inflammation using different commercially sourced SAA2-specific antibodies consistently showed (Liu et al., 2022a). To validate the specificity of placental SAA2 expression, we employed recombinant mouse SAA2 protein as a positive control. The anti-SAA2 antibodies, but not those against SAA1, SAA3 and SAA4, detected identical bands in the SAA2 positive control and IL-1β-treated placentas (Supplementary Figure S1B).

We utilized liquid chromatography mass-spectrometry (LC-MS) to quantify proteomic profiling and isoforms of SAA1/2 in the mouse placenta (Supplementary Figure S2A). Out of 7970 proteins identified and quantified, 142 proteins (1.79%) showed significant changes during inflammation (Supplementary Figure S2B). Proteins with a fold change in expression (IL-1β vs. PBS) greater than 25% are listed in Supplementary Figure S2C. To identify the isoform expression of SAA1 and SAA2 in the placenta, we collected all published amino acid sequences, including validated SAA1 (NP_033143, P05366), SAA2 (NP_035444, P05367) and their variants from different mouse strains for comparison (Supplementary Figure S2D) (Uhlar and Whitehead, 1999; Upragarin et al., 2005; Mori et al., 2014a; Foyn Bruun et al., 1998; Lowell et al., 1986; de Beer et al., 1993; Uhlar et al., 1994; Cathcart et al., 1996; Mori et al., 2014b; Patke et al., 2012). In the whole SAA protein, we detected a total of six trypsin-digested peptide fragments in the range of SAA 58-122 (GNYDAAQR (58-65), GPGGVWAAEK (66-75), ESFQEFFGR (81-89), EAFQEFFGR (81-89), GHEDTMADQEANR (90-102) and DPNYYRPPGLPDKY (109-122) shared by both SAA1 and SAA2. However, at the corresponding sites, highlighted in grey, the SAA1-specific fragments EGFQEFFGR (81-89) and GHEDTIADQEANR (90-102), and SAA2-specific fragment DPNYYRPPGLPAKY (109-122), were not detected. All detected tryptic peptides were shared between SAA1 and SAA2 isoforms, and therefore this proteomic approach could not reliably distinguish between the two due to their high sequence homology.

### Maternal administration of siSaa2 reduces placental SAA2 expression following sub-chronic maternal inflammation

**FIGURE 2:**
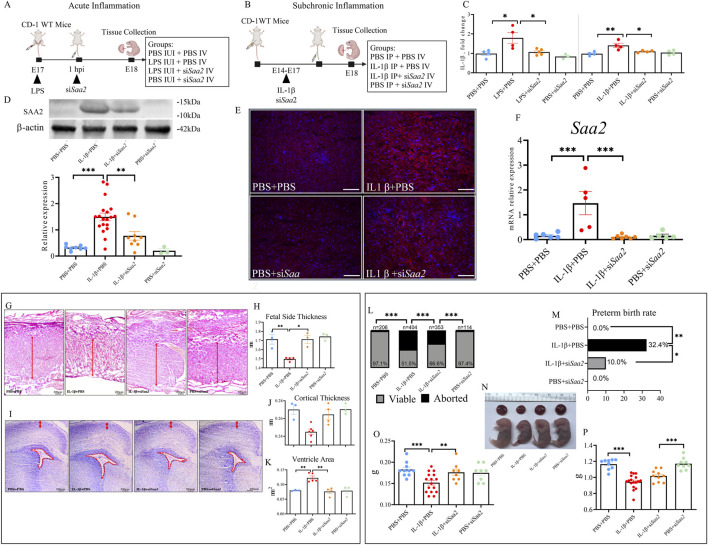
Maternal administration of si*Saa2* reduces placental SAA2 expression and alleviates abnormal placental histology and fetal brain cortical changes following sub-chronic maternal inflammation. **(A)** A schematic diagram illustrating the study design for the mouse model of acute intrauterine injection using LPS. **(B)** A schematic diagram illustrating the study strategy for the mouse model of sub-chronic maternal inflammation using IL-1β intraperitoneal injection. SiRNA was infused using intravenous injections. **(C)** Placental concentrations of IL-1*β* were measured by enzyme linked immunosorbent assay (ELISA). (*n =* 4 per group). **(D)** Representative Western blotting of Saa2 expression in the placenta and statistical analysis normalized to the levels of β-actin as a loading control. PBS+PBS, *n =* 8; IL-1*β*+PBS, *n* = 19; IL-1*β*+si*Saa2*, *n* = 9; PBS+si*Saa2*, *n* = 3. **(E)** Representative fluorescence immunostaining for Saa2 (red) and DAPI (blue, nucleic staining) in the labyrinth of placentas. *n* = 3, 5 images per mouse examined. Scale bars, 50 μm. **(F)** Real-time quantitative polymerase chain reaction (RT‒qPCR) was performed to evaluate the effect of siRNA treatment, *n =* 6 for each group **(G)** Hematoxylin and eosin (H&E) staining was performed to compare placental thicknesses at ×5 magnification. The solid arrows crossing the central cut surface of the placentas indicate the location of measurements: fetal segments were identified as a compilation of the junctional zone and labyrinth. Scale bars, 200 μm **(H)** Statistical analysis shows the results of H&E staining measuring fetal placental length at the thickest location. PBS+PBS, *n =* 3; IL-1*β*+PBS, *n =* 4; IL-1*β*+si*Saa2*, *n =* 3; PBS+si*Saa2*, *n =* 3. **(I)** Nissl staining was performed to measure cortical thickness (upper red arrows) and ventricle area (lower red panels) in the fetal brain. Scale bars, 200 μm. **(J,K)** Nissl staining measuring cortical thickness and ventricle area. Quantitative data, with the median indicated by the solid line for each group, represents the average of these measurements from five random brain sections for each litter. PBS+PBS, *n =* 3; IL-1*β*+PBS, *n =* 6; IL-1*β*+si*Saa2*, *n =* 4; PBS+si*Saa2*, *n =* 3 **(L)** The fetal viability per litter. **(M)** Preterm birth rates. PBS+PBS, *n =* 18; IL-1*β*+PBS, *n =* 34; IL-1*β*+si*Saa2*, *n =* 30; PBS+si*Saa2*, *n =* 10. **(N)** Representative images of the placenta and its corresponding fetus between groups. **(O,P)** Placenta **(O)** and fetal weights **(P)**. PBS+PBS, *n =* 10; IL-1*β*+PBS, *n =* 16; IL-1*β*+si*Saa2*, *n =* 8; PBS+si*Saa2*, *n =* 8. IUI: intrauterine injection; IV: intravenous; IP: intraperitoneal. Values are expressed as the mean ± SEM, One-way ANOVA with Bonferroni *post hoc* tests for multiple comparisons of normally distributed data and Kruskal‒Wallis with Dunn’s multiple comparisons tests for nonparametric data **(C,D,F,H,J,K,O,P)**. Chi-square test **(L,M)**, *p < 0.05, **p < 0.01, ***p < 0.001.

Chronically produced IL-1β is considered a link between placental immunity and associated inflammation (Chudnovets et al., 2020). Non-human primates injected with a single dose of IL-1β all delivered preterm within 48 h, while repeated injections of other cytokines are required to reproduce the same result (Sa et al., 2006), and similar effects have been observed in mouse models (Romero et al., 1991; Yoshimura and Hirsch, 2005). Studies have shown that endogenous IL-1β and SAA may interact reciprocally, potentially forming a vicious circle (Conti et al., 1995; Lachmann et al., 2009; Migita et al., 2014; Chen et al., 2023; Facci et al., 2018; Yu, 2023). Thus, IL-1β expression levels and the effect of the siRNA administration were examined. In both LPS- (Liu et al., 2022a) (Figure 2A) and IL-1β-induced (Figure 2B) inflammation mouse models, maternal treatment with si*Saa2* resulted in a significant reduction in the production of placental IL-1β (*p <* 0.05, Figure 2C), suggesting that siRNA effectively inhibited inflammatory IL-1β expression, which is highly associated with PTB.

Validation results showed that si*Saa2* treatment significantly reduced the placental expression of SAA2 (*p <* 0.01, Figure 2D) induced by sub-chronic inflammation. Immunofluorescence staining of SAA2 in the placenta (labyrinth) demonstrated that the administration of IL-1β and si*Saa2* resulted in lower SAA2 expression (Figure 2E) as well as mRNA level (Figure 2F) compared to the IL-1β group.

### Maternal administration of siSaa2 promotes recovery of placental and fetal brain morphology and improves fetal outcomes following sub-chronic inflammation

Hematoxylin and eosin (H&E) and Nissl staining were applied to placentas and fetal brains, respectively, to assess changes in their morphology following sub-chronic inflammation at E18. The thickness of the fetal side of the placenta was reduced significantly in our sub-chronic inflammation model (*p <* 0.01). In contrast, when si*Saa2* was administered to pregnant dams in the IL-1β groups, placental thickness was normalized to the expected thickness (*p <* 0.01, Figures 2G,H). Of note, si*Saa2* alone did not result in placental morphologic differences between the PBS+si*Saa2* group and the PBS+PBS group, indicating that si*Saa2* has no negative growth effects in the absence of inflammation.

Exposure to sub-chronic maternal inflammation resulted in a significant reduction in the cortical thickness and a significant increase in ventricle size in fetal brains (*p <* 0.01, Figures 2I–K). Maternal si*Saa2* treatment led to normal fetal cortical thickness and ventricle areas (*p <* 0.01) in the pups from the IL-1β+si*Saa2* group (*p <* 0.01). Our data indicate that maternal treatment with si*Saa2* mitigates the adverse effects of sub-chronic inflammation on placental structural damage and promotes normal fetal brain development.

Maternal treatment with si*Saa2* markedly reduced the abortion rate induced by sub-chronic inflammation (*p <* 0.001, Figure 2L). PTB rates were also significantly decreased by si*Saa2* administration (*p <* 0.05, Figure 2M). We checked the placenta and fetal weight between groups (Figures 2N–P). Maternal inflammation significantly reduced the weights of placentas (Figure 2O, *p <* 0.001) and fetuses (Figure 2P, *p <* 0.001). Treatment with si*Saa2* significantly normalized placental weight (*p <* 0.01) but fetuses in the IL-1β + si*Saa2* remained smaller without significant difference in weight from controls, suggesting some rescue of fetal growth.

**FIGURE 3:**
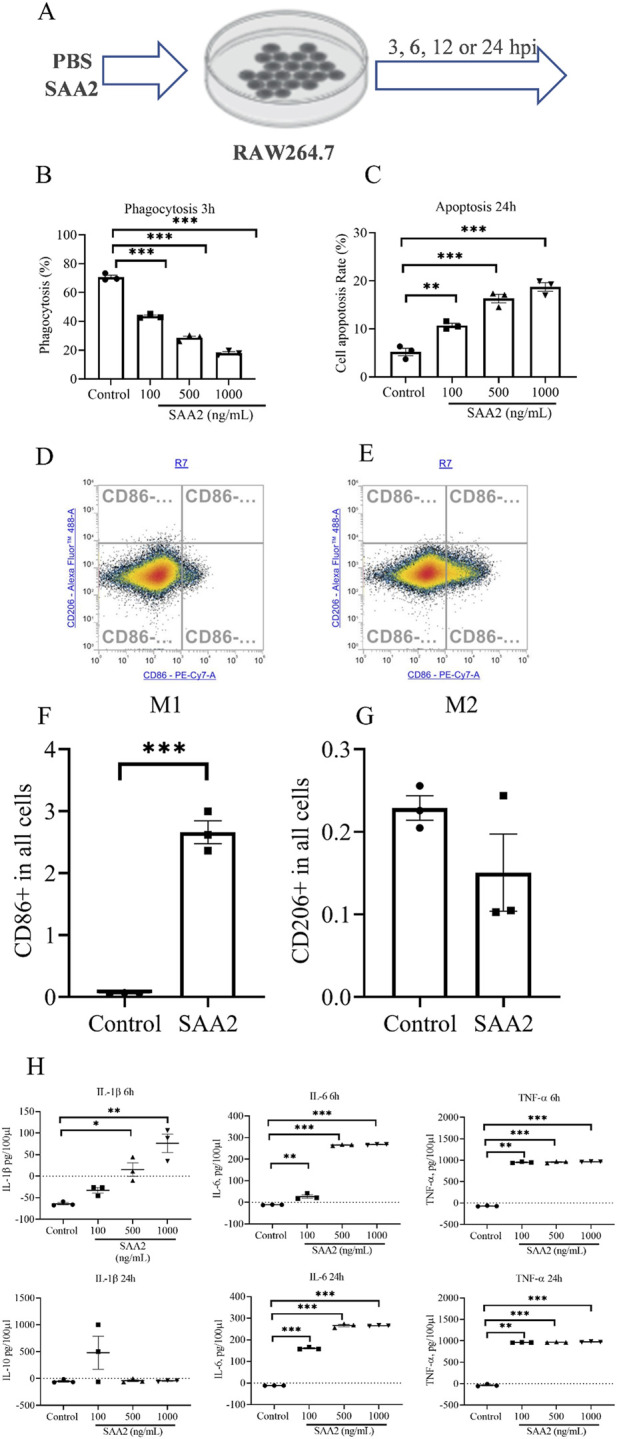
SAA2 induces inflammation in macrophage cells. **(A)** A schematic diagram illustrating our workflow involving recombinant mouse SAA2 (100 ng/mL, 500 ng/mL or 1 ug/mL) or PBS treatment of RAW264.7 macrophages for the indicated time periods (hpi = hours post-infection). **(B,C)** Cell phagocytosis and cytotoxicity as evaluated by flow cytometry and lactate dehydrogenase (LDH) assay, respectively. **(D–G)** Immune status as determined by flow cytometry **(D,E)** and their quantification **(F,G)**. **(H)** Concentrations of the indicated cytokines in the culture media and the time period at which they were measured by ELISA. Values are expressed as the mean ± SEM, *n* = 3 independent replicates performed at different time. One-way ANOVA One-way ANOVA with Bonferroni *post hoc* tests for multiple comparisons of normally distributed data, or Student’s t-test where appropriate. *p < 0.05, **p < 0.01, ***p < 0.001.

Given that SAA2 is absent in HBCs, we investigated the effect of extracellular SAA2 on macrophage using RAW264.7cells, a mouse macrophage cell line, exposed to increasing concentrations of recombinant SAA2 (Figure 3A). Phagocytotic capacity was significantly decreased in SAA2-treated cells at 3 h post-infection (hpi) (Figure 3B), while apoptosis was increased at 24 hpi (Figure 3C) in a dose-dependent manner. Flow cytometry analysis further revealed an increased proinflammation status (M1-like) of cells following SAA2 exposure (*p <* 0.001, Figures 3D–G). Consistently, te secretion of proinflammatory cytokines, including IL-1β, IL-6 and tumor necrosis factor (TNF)-α, was significantly elevatedin response to SAA2 treatment at either 6 or 24 hpi, or both (Figure 3H) Collectively, these data indicate that extracellular SAA2 acts on macrophages as effector cells to promote inflammatory dysfunction.

### Maternal administration of siSaa2 downregulates genetic placental inflammatory responses and other factors following sub-chronic inflammation

Using multiplexed immune arrays, we measured the placental mRNA expression of 96 immune-related genes and observed an increase in the expression of inflammatory cytokines *Il1β*, *Il6, Il15, Il5*, immune cell surface marker *Cd86* and *Ptprc*, and the transcription factor *Csf2* in response to maternal IL-1β exposure. siRNA treatment inhibited the expression of these genes (Supplementary Table S2). Individual RT-qPCR confirmed the reversal in expression of major cytokines *Il1β* and *Il6* induced by inflammation and siRNA treatment (Supplementary Figure S3).

### The P2X7 receptor is required to induce Saa2-associated preterm birth during sub-chronic inflammation

**FIGURE 4:**
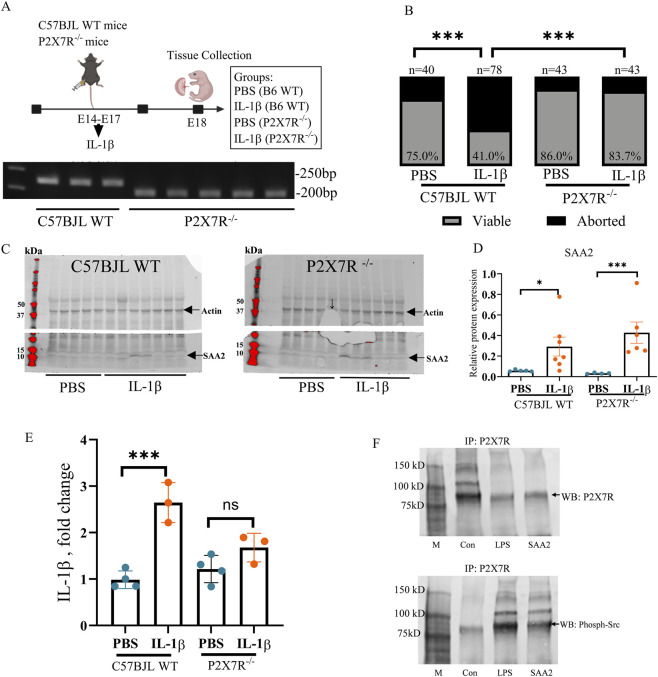
The P2X7 receptor is required for preterm birth induced by sub-chronic maternal inflammation. **(A)** A schematic diagram illustrating the study design. At E14-17, wild-type C57BJL (WT) and *P2x7r* knockout (P2X7R KO) mice underwent intraperitoneal injection (IP) with mouse recombinant IL-1*β* or PBS for four consecutive days. Placenta was harvested from dams at embryonic (E) day 18. The Agarose gel at the bottom panel demonstrates multiplex PCR-based genotyping of genomic DNA samples for the detection of *P2x7r* (+/+) and *P2x7r* (−/−) genotypes. **(B)** At E18, fetal viability was determined as the percentage of fetuses that were viable. WT groups: PBS, *n =* 40 from 5 dam; IL-1*β*, *n =* 78 from 11 dam; *P2x7r* ^*−/−*^ groups: PBS, *n =* 43 from 6 dam; IL-1*β*, *n =* 43 from 7 dam. **(C,D)** Gel images of SAA2 expression in the placenta and statistical analysis normalized to the levels of actin as a loading control. WT groups: PBS, *n =* 5; IL-1*β*, *n =* 7; P2X7R^−/−^ groups: PBS, *n =* 4; IL-1*β*, *n =* 6. One sample from the control P2X7R^−/−^ group was excluded from analysis because the band was not fully resolved, as indicated by the vertical arrow. **(E)** Placental IL-1β expression following maternal inflammation as determined by ELISA. WT groups: PBS, *n =* 4; IL-1*β*, *n =* 3; *P2x7r* ^*−/−*^ groups: PBS, *n =* 4; IL-1*β*, *n =* 3. **(F)** Immunoprecipitation followed by Western blot analysis of RAW264.7 cells exposed to inflammatory conditions. IP: immunoprecipitation. Values are expressed as the mean ± SEM, Chi-square test **(B)** and Student’s t-test **(D,E)**, *p < 0.05, **p < 0.01, ***p < 0.001.

SAA can bind to the P2X7 receptor (P2X7R), a ligand-gated cation channel, primarily expressed on macrophages (Collo et al., 1997; Dubyak, 2012) that triggers the release of IL-1β to induce proinflammatory effects (Sack, 2018; Di Virgilio et al., 2017). To evaluate whether P2X7R is involved in the effects of SAA2 on adverse fetal outcomes, *P2x7r* knockout (P2X7R KO) and wild-type (WT) mice subjected to timed pregnancy were treated with IL-1β (Figure 4A). In the WT groups, IL-1β-exposed pregnancies had significantly reduced pup viability compared to PBS-treated controls (*p <* 0.001, Figure 4B). However, in the P2X7R KO animals, pup viability was similar between controls and IL-1β-treated animals. This improvement was significant when the IL-1β-treated WT and P2X7R KO groups were compared (*p <* 0.001, Figure 4B). At E18, greater expression of Saa2 was observed in the WT (*p <* 0.05) and P2X7R KO (*p <* 0.001, Figures 4C,D) groups that received IL-1β, indicating a role for inflammatory signaling, with improved fetal outcomes in the receptor KO. The observed variability likely reflects biological heterogeneity among animals, together with minor variability introduced during placental tissue homogenization and protein extraction. IL-1β expression in the placenta was significantly higher in the WT, but not in the P2X7R KO, sub-chronic inflammation mouse model compared to the control (*p <* 0.001, Figure 4E), suggesting a role of P2X7R in mediating SAA-induced IL-1β production and abortion.

P2X7R contains intracellular domains capable of recruiting signaling kinases, including members of the Src family (Nie et al., 2021). Upon activation, P2X7R induces phosphorylation of Src at Tyr416, which contributes to IL-1β production and release (Alenghat et al., 2012). To model macrophage response to placental-derived extracellular SAA2 observed *in vivo*, RAW264.7 macrophages were exposed to recombinant SAA2 *in vitro*. Immunoprecipitation and Western blot analyses performed in RAW264.7 cells revealed that stimulation with either LPS or SAA2 increased the levels of phosphorylated Src (Tyr416) associated with P2X7R compared with control conditions (Figure 4F), supporting a mechanism whereby extracellular SAA2 activates P2X7R–Src signaling in macrophages.

### Distinct expression patterns of SAA1 and SAA2 in the human placenta

**FIGURE 5:**
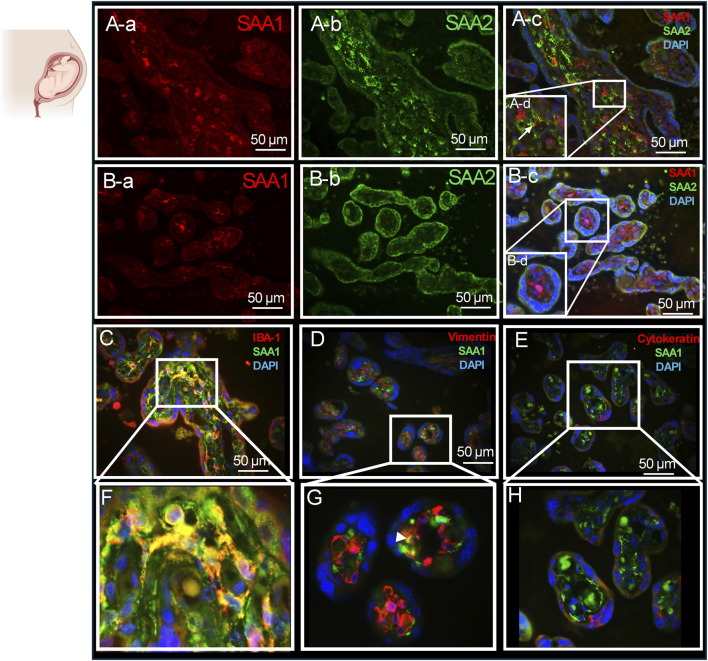
Expression patterns of SAA1 and SAA2 in the human term placenta. **(A)** Representative images of SAA1 and SAA2 expression in the stem villi. **(B)** Representative images of SAA1and SAA2 expression in the terminal villi. **(C,F)** Representative images of SAA1 and macrophage marker, IBA-1expression in the stem villi. **(D,G)** Representative images of SAA1 and endothelial marker, vimentin expression in the terminal villi. **(E,H)** Representative images of SAA1 and trophoblast marker, cytokeratin expression in the terminal villi. *n* = 5 placentas, 6 images per placenta examined, Scale bars: 10 μm for insect images; 50 μm for all other images.

We used IHC to differentiate SAA1 and SAA2 expressions in the villi of human placentas (Figure 5). SAA1 displayed homogenous expression in the cytoplasm, while SAA2 appeared in a punctate pattern within both the cell membrane and cytoplasm. At the stem villi level (Figure 5A), SAA1 was primarily expressed in the stroma (Figure 5A-a) whereas SAA2 was localized in the syncytiotrophoblast layer (the outer layer) as well as within the villi (Figure 5A-b). Although there was no clear overlap between SAA1 and SAA2 within the villi, they appeared to be highly associated (Figures 5A-c, d, arrow). At the level of terminal villi (Figure 5B), SAA1 maintains a similar expression pattern to that seen in the stem villi, remaining confined to the stroma (Figure 5B-a). In contrast, SAA2 was highly expressed in mature syncytiotrophoblasts but exhibited low expression within the villi (Figure 5B-b), resulting in a weaker association between SAA1 and SAA2 at this level (Figures 5B-c,d).

SAA1 expression was analyzed in combination with specific cell markers (Figures 5C–H). SAA1 was highly expressed in IBA-1-positive macrophages within the villi (Figures 5C,F) and showed mild colocalization with vimentin-positive endothelial cells (Figures 5D,G, arrowhead). However, SAA1 was not expressed in trophoblast cells (Figures 5E,H). Our immunofluorescence staining data comparing mouse and human tissues revealed that, despite sharing the same terminology, SAA1 and SAA2 exhibit distinct expression patterns across tissue types, cell types, and species.

## Discussion

Using a mouse model which recapitulates clinical scenario of inflammation-induced preterm birth in the second and third trimester of human pregnancy, our study demonstrates that maternal treatment with an siRNA targeting *Saa2,* reduces *Saa2* mRNA and SAA2 protein expression*,* providing therapeutic benefits for placental inflammation and adverse neonatal outcomes induced by intrauterine inflammation.

In this study, we utilized recombinant mouse IL-1β, mimicking the endogenous mediator of the inflammation cascade (Chudnovets et al., 2020), at moderate doses with prolonged inflammation and treatment duration (Sones and Davisson, 2016). In contrast to our previous studies using LPS intrauterine injection to mimic preterm birth, where immune array analysis was characterized mainly by increased levels of *Ccl2*, *Ccl3*, *Cxcl9*, and *Cxcl10* (Novak et al., 2018), the profiles observed in the IL-1β-mediated model used in the current study differed significantly, showing increased levels of *Il6, Il15, Il5*, *Cd86, Ptprc* and *Csf2* except for the elevation of IL-1β itself. Even the route of IL-1β delivery (intrauterine vs*.* systemic) influenced the immune response in the placenta (Lee et al., 2019), highlighting the complexity of immune responses within the maternal environment. Given that SAA is typically recognized as an acute inflammatory marker, it is crucial to carefully identify its role using an appropriate animal model for therapeutic purposes and mechanistically determine its relationship to IL-1β secretion.

P2X7R and Src family kinases (SFKs) are functionally linked in bidirectional regulation in several signaling contexts. The P2X7 receptor, a trimeric ATP-gated ion channel, is highly expressed in macrophages. SFKs are non-receptor tyrosine kinase that mediate signal transduction downstream of immune receptors upon phosphorylation at Tyr416 (p-Src) (Hedden et al., 2011). Study has demonstrated that ATP stimulation induces a conformational change in P2X7R, enabling Src to physically associate with the receptor’s cytoplasmic tail (Kopp et al., 2019) or with nearby adaptor proteins (Alenghat et al., 2012). Once recruited, Src becomes activated and phosphorylates downstream substrates, such as NF-κB signaling, promoting inflammatory gene expression, driving inflammasome activation (Singer et al., 2011) and cytokine release (Valimaki et al., 2013). SAA has been shown to stimulate APT release in macrophages (Chen et al., 2019) ^70^. Consistent with this, our findings support a model in which placental-derived extracellular SAA2 acts on macrophages as effector cells via P2X7R-dependent signaling, contributing to inflammatory dysfunction during pregnancy.

The improvement in fetal viability observed in P2X7R-deficient mice despite persistent placental Saa2 expression suggests that the detrimental effects of SAA2 are mediated primarily through downstream P2X7R signaling rather than Saa2 abundance alone. This supports a model in which P2X7R acts as a key effector translating extracellular SAA2 signals into pathogenic inflammatory responses. At the same time, the partial uncoupling between Saa2 expression and fetal outcome in the knockout background indicates that compensatory or parallel inflammatory pathways may contribute when P2X7R signaling is absent. Together, these findings highlight the central role of the SAA2–P2X7R axis while acknowledging the involvement of additional modulatory mechanisms.

Distinct from prior studies in non-pregnant species, where SAA was thought to be majorly produced by liver parenchymal cells (Uhlar and Whitehead, 1999), we found that SAA isoforms are also expressed in placenta in both mice and human. We delivered siRNA via an IV route, as animal origin-free lipid nanoparticle-based technology favors delivery into tissues with high blood flow (Chudnovets et al., 2020; Sajid et al., 2020; Biscans et al., 2021) and that the placenta is a highly vascularized organ. The current study, combined with our previous data targeting placental *Saa2* in mice rather than in other organs, supports the use of IV infusion of siRNA as a means of delivering therapeutics to downregulate placental *Saa* expression in mice.

There is a notable specific species discrepancy that exists in SAA isoforms. Based on chromosomal mapping, it appears that the mouse *Saa2* locus corresponds to human *SAA1.* The Nomenclature Committee of the International Society of Amyloidosis has recommended that the mouse nomenclature be fully compatible with human nomenclature. Thus, “mouse *Saa1* was originally described as *Saa2* and/or that mouse *Saa2* was originally described as *Saal* as necessary” (Uhlar and Whitehead, 1999; Sipe, 1999) as claimed by the Committee. This could lead to confusion regarding the SAA term in publication and production. However, the urgency of developing new therapeutic targets and approaches for preterm birth cannot be delayed by the complexity of nomenclature.

While SAA are relatively small proteins with well-documented sequences and polymorphisms, their three-dimensional structures have remained elusive due to their poor solubility (Sack, 2018). Differences in their spatial structures could impact their roles in inducing PTB and abortion during maternal inflammation. Additionally, minor sequence variations may lead to differences in protein interactions, likely contributing to the distinct role of SAA isoforms in the placenta. Studies have shown that Saa2 in the CE/J mouse, which is a composite of Saa1 and Saa2 of C57BL/6J strain, demonstrates resistance to amyloid A protein amyloid formation (Sack, 2018; Mori et al., 2014b), contrast with the typical SAA functions of tissue deposition.

Our previous study used multiple Saa1, and 2 antibodies targeting mature protein and identified distinct expression patterns in both mouse and human placentas. However, the possibility of cross-reactivity cannot be entirely ruled out. We have utilized mass spectrometry, but we could not distinguish the unique sequence of Saa1 and Saa2 in mice, respectively, likely due to the technical limitations and the stability and post-translational modification of peptides in placental tissues. Further clarification and improvement, including peptide-targeted monoclonal antibodies and advanced mass spectrometry techniques, are needed.

It should be noted that SAA isoforms differ markedly on expression patterns between mouse and human, and the limited number of human term placental samples analyzed in this study constrains the generalizability of our findings across gestational stages and pathological conditions. In future work, we aim to expand our placental tissue collection to include samples spanning a broader range of gestational ages, as well as clinically relevant conditions such as preterm birth. This approach will enable more comprehensive validation and allow our findings to be extended to diverse physiological and pathological contexts.

Although systemic siSaa2 administration improved placental and fetal outcomes, direct assessment of siRNA biodistribution, placental cell–type–specific uptake, and off-target effects was not performed. Future studies will incorporate biodistribution analyses and placenta-targeted delivery strategies, including nanoparticle- and ligand-mediated approaches, to enhance placental specificity. In addition, long-term maternal and postnatal safety was not evaluated and will be addressed through planned assessments of maternal health, postnatal growth, neurodevelopmental outcomes, and toxicity. Further *in vivo* interrogation of placental signaling pathways, particularly in a cell-type–specific context, will also be required to refine the mechanistic framework.

The results presented here contribute to a better understanding of the interplay between SAA inhibition and placental immune regulation, aiding in the development of new therapeutic strategies to reduce preterm birth induced by maternal inflammation and its adverse perinatal sequelae. Moving forward, the siRNA technique could identify additional drug candidates with more targeted actions suitable for use during pregnancy.

## Data Availability

The data presented in this study are deposited in the PRIDE repository, accession number PXD072875.
