# Supplementary material for: Targeting SAA expression via siRNA mitigates preterm birth induced by maternal inflammation
Source: Front Pharmacol. 2026 Jan 16;17:1749966. doi: 10.3389/fphar.2026.1749966 (PMC12855142; doi:10.3389/fphar.2026.1749966)
Supplement: Supplementary file 1 [file DataSheet1.pdf]

A

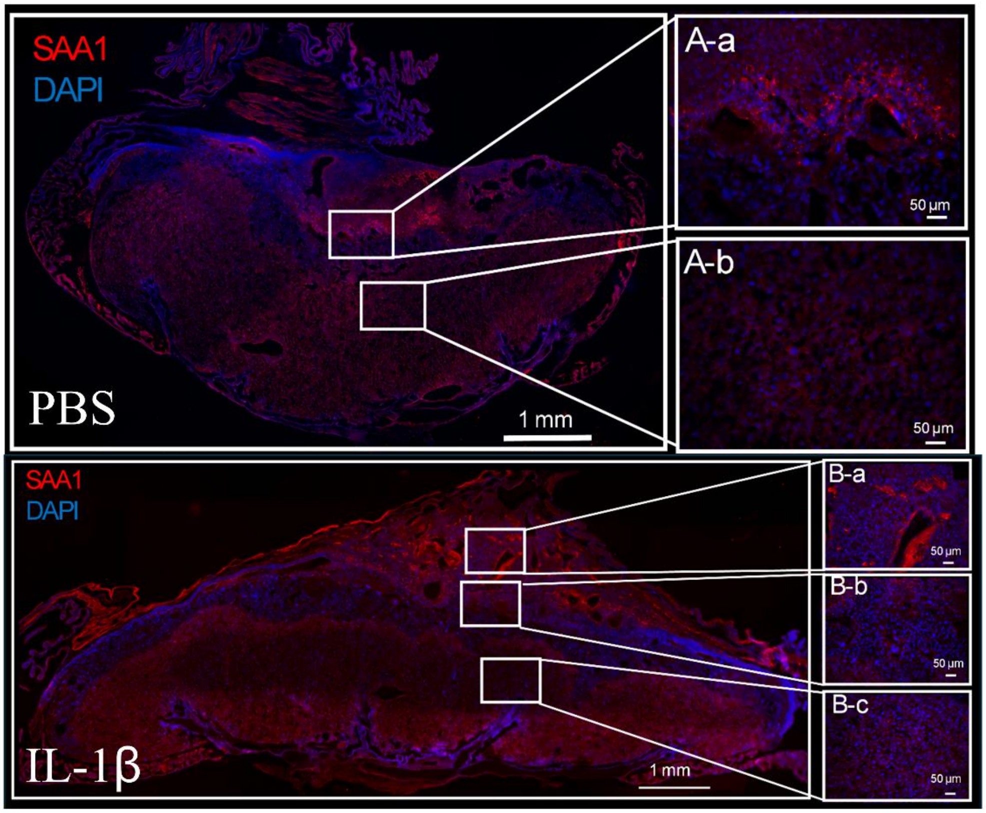
SAA1
DAPI
A-a
50 µm
A-b
50 µm
PBS
1 mm
SAA1
DAPI
B-a
50 µm
B-b
50 µm
B-c
50 µm
IL-1β
1 mm

B

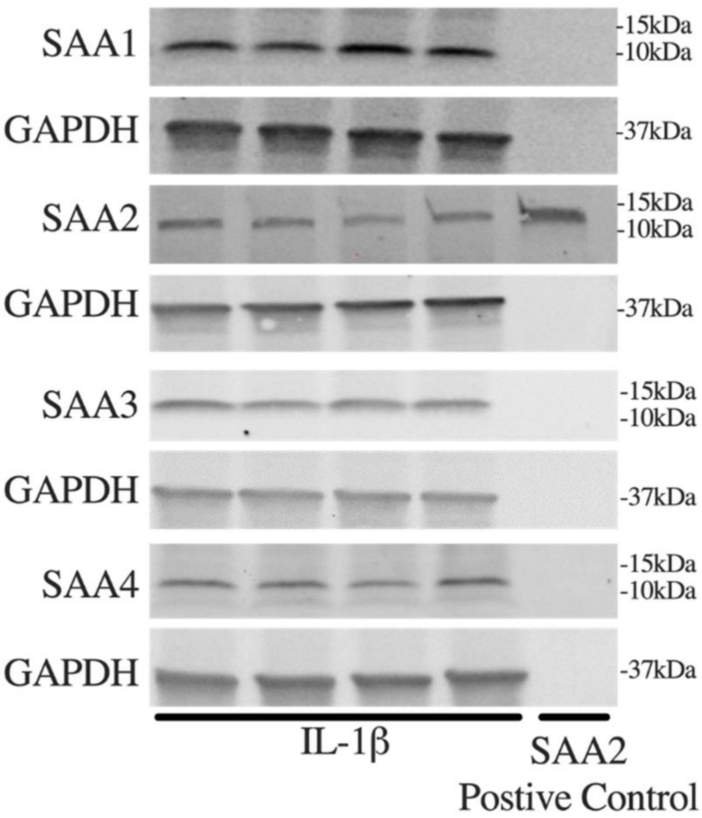
SAA1
-15kDa
-10kDa
GAPDH
-37kDa
SAA2
-15kDa
-10kDa
GAPDH
-37kDa
SAA3
-15kDa
-10kDa
GAPDH
-37kDa
SAA4
-15kDa
-10kDa
GAPDH
-37kDa
IL-1β
SAA2
Postive Control

Figure S1. SAA1 expression pattern in the mouse placenta.
(A) Representative images of SAA1 expression in the PBS and IL-1β groups (*n* = 3, 5 images per mouse examined). Scale bar in left panel, 1mm; in right panel, 50 µm. **(B)** Gel identification of Saa2 using a positive control of mouse Saa2 protein (n = 4).

The bottom-left image represents the non-primary control.

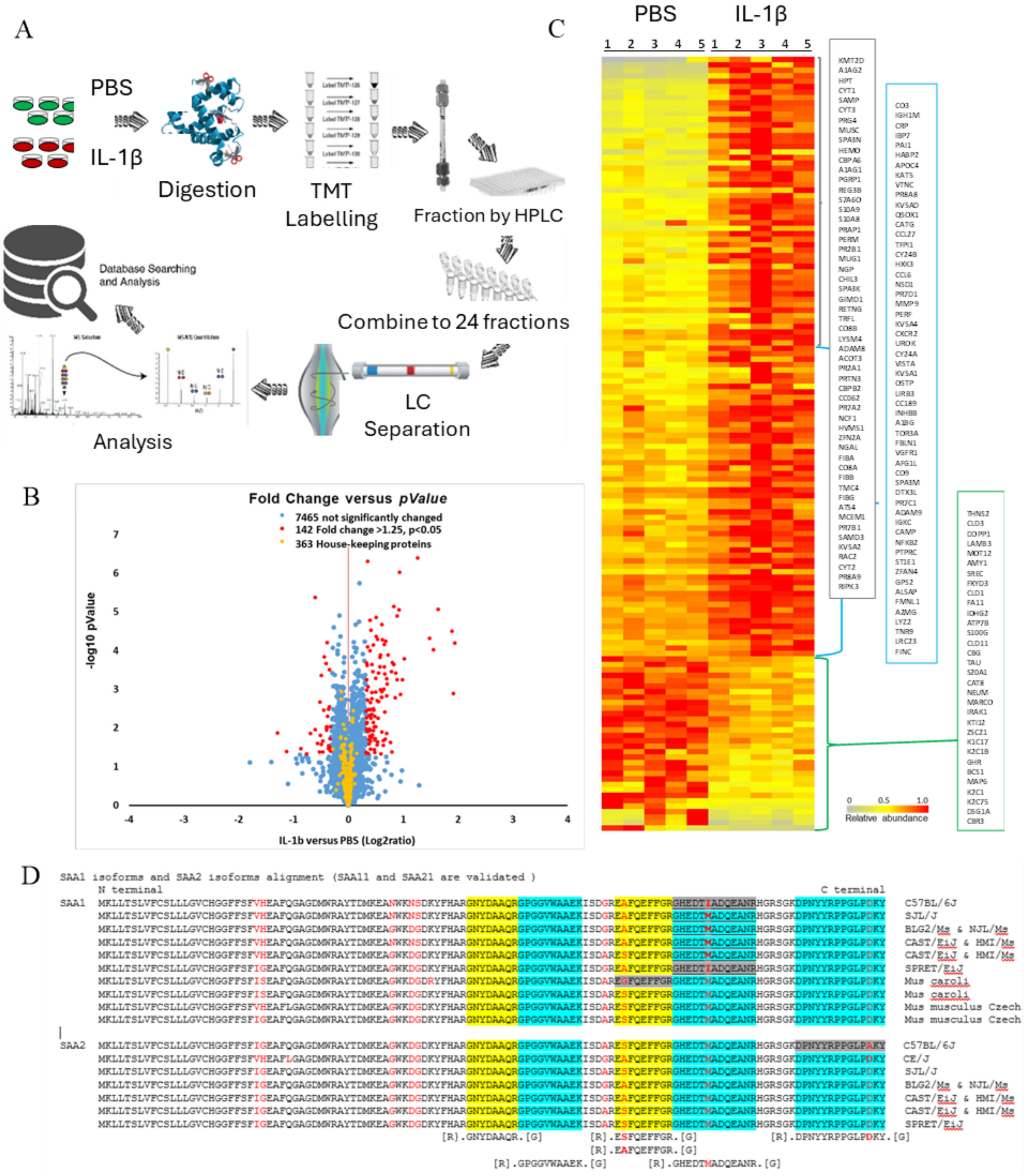

Figure S2. Mass spectrometry analysis of mouse placenta.

(A) A schematic diagram illustrating the mass spectrometry-based quantitative proteomic profiling workflow of our mouse samples**. (B)** Proteomic characterization of IL-1β-treated mouse placenta. **(C)** Profiling of proteins with statistical significance (n = 5 for each group). **(D)** Isoforms of Saa1 and Saa2 in the indicated mouse strains.

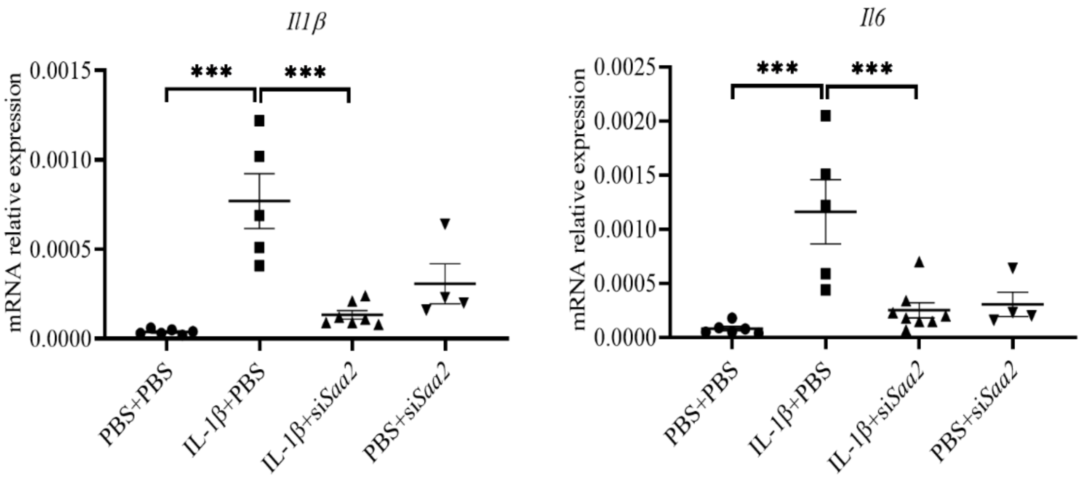

Figure S3. Confirmatory real-time quantitative PCR.

From embryonic day (E) 14 to E17, CD-1 mice received intraperitoneal (IP) injections of recombinant mouse IL-1β for four consecutive days. One hour after each injection, dams were administered an intravenous injection of either siRNA targeting serum amyloid A 2 (si*Saa2*) or phosphate-buffered saline (PBS) daily. Placentas were collected for immune array analysis and real-time qPCR. Individual confirmatory real-time quantitative PCR was performed for the major inflammatory cytokines IL-1β and IL-6, following the immune array assay (Supplemental Table 2). Values are expressed as the mean ± SEM, PBS+PBS, $n = 5$; IL-1β+PBS, $n = 5$; IL-1β+siSaa2, $n = 7$; PBS+siSaa2, $n = 4$, One-way ANOVA with Bonferroni post hoc tests for multiple comparisons of normally distributed data and Kruskal–Wallis with Dunn's multiple comparisons tests for nonparametric data. ***$p < 0.001$

Supplemental Table 1 Antibodies for immunofluorescence staining, flow cytometry and Western blot

**Primary antibodies**	**Clone**	**Manufacture**	**Catalog No.**
SAA1		Abcam	Ab171030
SAA1		Antibodies Online	ABIN7193595
SAA1		Antibodies Online	ABIN7118966
SAA2		Biomatik	CAU25293
SAA2		Biomatik	CAU25292
SAA3		Mybiosource	MBS8548603
SAA4		Mybiosource	MBS3206295
IBA-1	EPR16588	Abcam	ab178846
Vimentin	RV202	Abcam	ab8978
Cytokeratin	C-11	Abcam	ab7753
F4/80	CI: A3-1	Abcam	ab6640
CD 86	GL1	Invitrogen	25-0862-82
CD206	MR6F3	Invitrogen	53-2061-82
P2X7R		Proteintech	28207-1
phospho-Src family (Tyr416)		Cell Signaling	2101
**Secondary antibodies**			
Alexa Fluor 488 Donkey anti-rabbit		Invitrogen	A21206
Alexa Fluor 568 Donkey anti-mouse		Invitrogen	A10037
Alexa Fluor 488 Goat anti-mouse		Invitrogen	A11001
Alexa Fluor 488 Goat anti-rat		Invitrogen	A11006
Alexa Fluor 568 Goat anti-rabbit		Invitrogen	A11036
Alexa Fluor 647 Donkey anti-rabbit		Invitrogen	A31573
